# Impact of early daycare on healthcare resource use related to upper respiratory tract infections during childhood: prospective WHISTLER cohort study

**DOI:** 10.1186/1741-7015-12-107

**Published:** 2014-06-26

**Authors:** Marieke LA de Hoog, Roderick P Venekamp, Cornelis K van der Ent, Anne Schilder, Elisabeth AM Sanders, Roger AMJ Damoiseaux, Debby Bogaert, Cuno SPM Uiterwaal, Henriette A Smit, Patricia Bruijning-Verhagen

**Affiliations:** 1 Julius Center for Health Sciences and Primary Care, University Medical Center Utrecht, STR 6.131, PO Box 85500, 3508 GA Utrecht, The Netherlands; 2 Department of Otorhinolaryngology, Division Surgical Specialties, Wilhelmina Children’s Hospital, University Medical Center Utrecht, 3508 AB Utrecht, The Netherlands; 3 Department of Paediatric Pulmonology, Wilhelmina Children’s Hospital, University Medical Center Utrecht, 3508 AB Utrecht, The Netherlands; 4 Department Paediatric Immunology, Wilhelmina Children’s Hospital, University Medical Center Utrecht, 3508 AB Utrecht, The Netherlands; 5 evidENT, Ear Institute, University College London, WC1X8EE London, UK; 6 National Institute for Public Health and the environment (RIVM), 3720 BA Bilthoven, The Netherlands

**Keywords:** Upper respiratory infection, Otitis media, Daycare, Healthcare utilization, Paediatric, Cohort study

## Abstract

**Background:**

Daycare attendance is an established risk factor for upper respiratory tract infections (URTI) and acute otitis media (AOM). Whether this results in higher use of healthcare resources during childhood remains unknown. We aim to assess the effect of first year daycare attendance on the timing and use of healthcare resources for URTI and AOM episodes during early childhood.

**Methods:**

In the Wheezing-Illnesses-STudy-LEidsche-Rijn birth cohort, 2,217 children were prospectively followed up to age six years. Children were categorized according to first-year daycare attendance (yes versus no) and age at entry when applicable (age 0 to 2 months, 3 to 5 months and 6 to 12 months). Information on general practitioner (GP) diagnosed URTI and AOM, GP consultations, antibiotic prescriptions and specialist referral was collected from medical records. Daycare attendance was recorded by monthly questionnaires during the first year of life.

**Results:**

First-year daycare attendees and non-attendees had similar total six-year rates of GP-diagnosed URTI and AOM episodes (59/100 child-years, 95% confidence interval 57 to 61 versus 56/100 child-years, 53 to 59). Daycare attendees had more GP-diagnosed URTI and AOM episodes before the age of one year and fewer beyond the age of four years than non-attendees (*P*_interaction_ <0.001). Daycare attendees had higher total six-year rates for GP consultation (adjusted rate ratio 1.15, 1.00 to 1.31) and higher risk for specialist referrals (hazard ratio: 1.43, 1.01 to 2.03). The number of antibiotic prescriptions in the first six years of life was only significantly increased among children who entered daycare between six to twelve months of age (rate ratio 1.32, 1.04 to 1.67). This subgroup of child-care attendees also had the highest overall URTI and AOM incidence rates, GP consultation rates and risk for specialist referral.

**Conclusions:**

Children who enter daycare in the first year of life, have URTI and AOM at an earlier age, leading to higher use of healthcare resources compared to non-attendees, especially when entering daycare between six to twelve months. These findings emphasize the need for improved prevention strategies in daycare facilities to lower infection rates at the early ages.

## Background

Daycare attendance is associated with increased incidence of respiratory infections at preschool age and it has been suggested that the incidence simply shifts to earlier ages such that the overall incidence remains similar. However, respiratory infections early in life might lead to a higher use of healthcare resources.

Upper respiratory tract infections (URTIs) and acute otitis media (AOM) are the most common reasons for doctor consultations and antibiotic use in children. The primary care incidence of URTI, including AOM, in children up to four years of age is estimated at approximately 400 per 1,000 child-years [1,2]. These infections have a significant impact on the child and on family life and carry a considerable economic burden [3-7].

Daycare attendance is a well-established risk factor for URTIs and AOM in preschool children [8-13]. Early life daycare attendance, however, has been suggested to protect against the common cold and ear infections beyond the preschool years [14-16]. Although this implies that daycare attendance influences the timing of infections rather than the overall number of infections a child experiences during childhood, the risk of infection-related complications might be higher during infancy compared to childhood, due to immaturity of the immune system [17].

Previous studies showed higher general practitioner (GP) consultation and antibiotic prescription rates in daycare attendees [12,18-22]. Furthermore, daycare attendance leads to higher specialist referral rates in children under the age of two years [23]. Whether this increased use of healthcare resources among daycare attendees at preschool age is offset by a lower use of healthcare resources beyond preschool age in the same children remains to be determined.

The aim of this cohort study, therefore, is to assess the long-term effect of first-year daycare attendance on the number and timing of GP diagnosed URTI and AOM episodes and total use of healthcare resources up to six years of age.

## Methods

### Whistler cohort study

This study was performed as part of the WHeezing and Illnesses STudy LEidsche Rijn (WHISTLER), a prospective birth-cohort study on perinatal and infant risk factors for wheezing illness. WHISTLER enrolled healthy newborns born between December 2001 and December 2012 living in the Leidsche Rijn district of Utrecht, The Netherlands. Study design and rationale of WHISTLER are described in detail elsewhere [24]. Briefly, parents of newborns were invited by telephone to participate within two months after birth. Exclusion criteria at baseline were gestational age <36 weeks, major congenital abnormalities and neonatal respiratory disease.

### Data collection

At baseline, data were collected on prenatal risk factors and parental characteristics. Data on postnatal risk factors, such as daycare attendance and duration of breastfeeding, were collected prospectively by monthly questionnaires during the first 12 consecutive months.

Follow-up of participants included extraction of six years of relevant medical data from the GP electronic medical database using the International Classification of Primary Care (ICPC) [25] and the Anatomical Therapeutical Chemical (ATC J01) coding systems. Specifically, we extracted data on URTI and AOM related GP consultations, antibiotic prescriptions and specialist referrals for respiratory infections for all participants with a GP practicing within the Leidsche Rijn research district. The paediatric medical ethics committee of the University Medical Center Utrecht approved the study. Written informed consent was obtained from the parents.

### Definition of outcomes

For the primary outcome variable we combined the number of GP-diagnosed URTI and AOM episodes during the first six years of life. URTI was defined as ICPC code R74 (acute URTI), R75 (sinusitis), R76 (tonsillitis/peritonsillar abscess), and/or R77 (acute laryngitis/tracheitis). AOM was defined as ICPC code H71 (AOM). A new episode of URTI and AOM was documented after a disease-free interval of at least 28 days. For each episode, we extracted additional data on number of GP consultations per URTI and AOM episode, related antibiotic prescriptions and specialist referrals. An antibiotic prescription or referral was considered to be related when dated from seven days before the start of an episode for URTI or AOM until seven days after the end of an episode.

### Definition of exposure and confounders

The exposure variables of interest were daycare attendance in the first year of life and age of entry, defined as the age at which a child first entered daycare for at least one half day per week in the first year of follow-up. Age of entry was categorised as: (1) no daycare in first year of follow-up (reference group); (2) start of daycare before three months of age; (3) from three to five months of age; and (4) six to twelve months of age. Additional infant characteristics, such as gender, parental education level as an indicator for socioeconomic status (SES), presence of older siblings and duration of exclusive breastfeeding, were considered as confounders. Educational level was categorised as high if one or both parents had completed at least vocational or university education and middle/low if both parents completed education lower than vocational or university level. Duration of exclusive breastfeeding was divided into four categories: no breastfeeding, one to three months, four to six months and more than six months of breastfeeding.

### Statistical analysis

Baseline characteristics of children according to first-year daycare attendance were compared using Chi-squared tests. For descriptive purposes, the incidence of URTI and AOM per 100 child-years was calculated by dividing the number of URTI and AOM episodes by the total number of child-years. Incidence was calculated per one-year age categories separately up to six years of age. The distribution of GP diagnosed URTI and AOM episodes, GP consultations for URTI or AOM and antibiotic prescriptions, were strongly over-dispersed. Therefore, we used generalized estimating equations (GEE) with a negative binomial link function to assess the association between first-year daycare attendance and the number of episodes of URTI and AOM with no first-year daycare as the reference group. The GEE method takes into account the correlation between repeated measurements in the same individual. To test whether the effect of first-year daycare on URTI and AOM was age-dependent, an interaction term for daycare attendance with age was included in the GEE model. The regression coefficients from the GEE model reflect an incidence rate ratio (IRR).

Next, we studied the associations between daycare attendance and cumulative number of GP consultations for URTI and AOM and antibiotic prescriptions for URTI and AOM in the first six years of life. We used generalized linear models with a negative binomial link function. GP follow-up duration was used as the offset variable to indicate exposure time. GP follow-up duration was estimated as the time from birth, as if they were registered by the GP within the first three months of life, or in case of a registry beyond the first three months of age, from time of GP registry until the last known date of follow-up or until their sixth birthday. The regression coefficients reflect rate ratios (RR).

Cox proportional hazard regression was performed to study the association between first-year daycare attendance and first URTI or AOM related specialist referral. GP follow-up duration was used as the underlying time metric. We censored individual observations either at the date of a first event, at the last known date of follow-up, or at the age of six years. The regression coefficients reflect a hazard ratio (HR), which should be interpreted as a relative risk.

All models were adjusted for potential confounders, including gender, parental education level, older siblings and duration of exclusive breastfeeding.

Because of missing values in parental- and child-related factors, a substantial proportion of the cases could not be used in the regression analysis. To address the potential bias incurred by using complete case-analysis, we imputed missing values using the multivariate imputation by chained equations (MICE) procedure in SPSS (version 20.0) [26]. The 10 imputed data sets were analysed and results combined. We averaged estimates of the variable to give a single mean estimate and adjusted standard errors according to the Rubin’s rule [27]. All statistical analyses were performed with SPSS version 20.0 (SPSS Inc, Chicago, IL, USA), SAS 9.2 (SAS Institute, Inc., Cary, NC, USA) and OpenEpi: Open Source Epidemiologic Statistics for Public Health (version 3.01, updated April 2013).

## Results

### Study population

**Figure 1:**
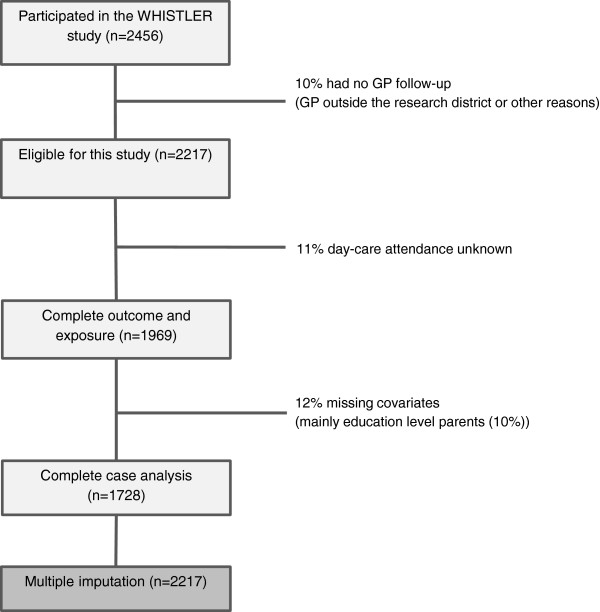
Flow chart of the study population.

During the entire WHISTLER recruitment period (December 2001 to December 2012), parents of 2,456 infants agreed to participate in this study. In April 2013, 2,217 infants (90%) had a GP inside the research district and were included in this analysis. Complete data were available for 1,728 of the 2,217 children (78%) (Figure 1).

Baseline characteristics of the 1,728 children in the total study population and according to daycare attendance are listed in Table 1. Children attending daycare in the first year of life had higher educated parents, less frequently had older siblings and more often received breastfeeding compared to children cared for at home. During the 7,543 person-years of follow-up (median 4.9, maximum 6.0 years) 4,243 episodes of GP diagnosed URTI and AOM were recorded (Table 1). Overall, the incidence rate of URTI and AOM was highest in the first two years of life and declined thereafter. Compared to children cared for at home, first-year daycare attendees had a higher incidence rate for URTI and AOM in the first year of life, but lower incidence rates at ages four to six years.

**Table 1:** Characteristics of children and parents according to entry in daycare

**Study population**	**Baseline population with complete data**	**No daycare in first year of life (ref)**	**Daycare entry in first year of life**	***P*-value**
	**Number = 1,728**	**Number = 350**	**Number = 1,378**	
**Parental characteristics**				
Parental education level (%)				<0.001
Low/middle	17.3	39.7	11.6	
High (at least one parent)	82.7	60.3	88.4	
**Child characteristics**				
Gender (% boy)	49.2	47.4	49.7	0.241
Duration of exclusive breastfeeding (%)				<0.001
No breastfeeding	18.9	27.4	16.8	
0 to 3 months	44.3	38.6	45.8	
4 to 6 months	22.8	17.4	24.2	
>6 months	13.9	16.6	13.3	
% with older siblings	51.6	58.9	49.7	0.001
IR of URTI and AOM per age category /100 child-years (95% confidence interval)				
0 to 1 year	92.9 (88.3 to 97.7)	59.1 (51.1 to 67.9)	100.4 (96.0 to 107.0)	<0.001
1 to 2 year	76.5 (72.2 to 80.8)	71.2 (63.0 to 81.4)	77.6 (72.9 to 82.6)	0.28
2 to 3 year	46.0 (42.5 to 49.7)	52.5 (44.7 to 61.2)	44.2 (40.4 to 48.3)	0.07
3 to 4 year	33.0 (29.7 to 36.4)	38.5 (31.5 to 46.7)	31.5 (28.0 to 35.3)	0.09
4 to 5 year	28.9 (25.7 to 32.5)	41.7 (33.9 to 50.8)	25.0 (21.6 to 28.8)	<0.001
5 to 6 year	25.5 (22.1 to 2.92)	41.6 (33.2 to 51.6)	20.4 (17.0 to 24.3)	<0.001

AOM, acute otitis media; IR, incidence rate; URTI, upper respiratory tract infection.

The following reported results pertain to the multiple imputation data sets (n = 2217).

### Association between number of episodes and daycare attendance

**Figure 2:**
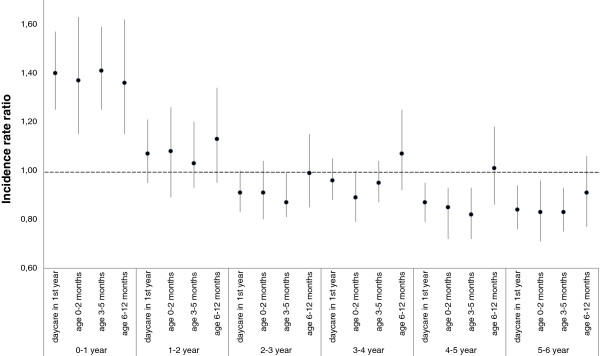
**The adjusted incidence rate ratios comparing the total number of URTI and AOM episodes at ages 0 to 6 years in children with and without day care in the first year of life and according to age at entry (no daycare in the first year is the reference group).** Adjustments were made for: gender, parental education level, older siblings and duration of exclusive breastfeeding. AOM, acute otitis media; URTI, upper respiratory tract infection.

Table 2 shows the number and incidence of GP-diagnosed URTI and AOM episodes during the first six years of life in children with and without first-year daycare attendance. After adjustment for gender, parental education level, presence of older siblings and duration of exclusive breastfeeding, the average six-year incidence was not significantly different between children who did and did not attend daycare. Test for interaction revealed that the association between first-year daycare attendance and URTI and AOM incidence was age-dependent (*P* <0.001). Additional analysis showed that after using any (partial or exclusive) breastfeeding as a confounder, instead of exclusive breastfeeding, the rate ratios remained similar for first-year daycare (data not shown).Figure 2 shows the adjusted incidence rate ratios for the outcomes at separate ages, 0 to 6 years, in children with and without first-year daycare and according to age at entry. During the first year of life the number of episodes was higher in the children attending daycare (adjusted IRR (aIRR): 1.40; 95% confidence interval (CI): 1.25 to 1.57) compared to children cared for at home. Between age one and four years differences were minimal and mostly non-significant. After preschool age (≥4 years of life in the Netherlands) the aIRRs reversed, showing lower IRRs in children attending daycare (aIRR age four to five years: 0.87; 95% CI: 0.80 to 0.95 and age five to six years: 0.85; 95% CI: 0.77 to 0.94). However, these lower incidence rates were not observed in children entering daycare from six months of age.

**Table 2:** Incidence of URTI and AOM during the first six years of life by daycare attendance

**GP diagnosed URTI and AOM by daycare attendance**	**Number of episodes**	**Child-years**	**Rate/100 child-years (95% CI)**	**Adjusted**^**a **^**IRR (95% CI)**
No daycare in first year	1,362	2,424.3	56.2 (53.3 to 59.2)	1.00
Day care in first year	4,334	7,325.2	59.2 (57.4 to 60.9)	1.08 (0.95 to 1.22)
Age 0 to 2 months	629	1,104.4	56.9 (52.7 to 61.4)	1.05 (0.88 to 1.25)
Age 3 to 5 months	2,967	5,081.0	58.4 (56.3 to 60.5)	1.06 (0.94 to 1.21)
Age 6 to 12 months	738	1,139.8	64.8 (60.3 to 69.5)	1.18 (1.00 to 1.40)

^a^Adjusted for: gender, parental education level, older siblings and duration of exclusive breastfeeding. AOM, acute otitis media; CI, confidence interval; GP, general practitioner; IRR, incidence rate ratio; URTI, upper respiratory tract infection.

### URTI and AOM related use of healthcare resources

Table 3 shows the use of healthcare resources due to GP-diagnosed URTI and AOM episodes during the first six years of life in children with and without first-year daycare attendance. The cumulative number of GP consultations for URTI and AOM was 15% higher (adjusted (a)RR: 1.15; 95% CI 1.00 to 1.31) in the group attending daycare. Similarly, the risk for URTI or AOM related specialist referral in the first six years of life was increased in first year daycare attendees (aHR: 1.43; 95% CI: 1.01 to 2.03). Overall, the increase in number of GP consultations and risk for specialist referral was most pronounced in children entering daycare between six to twelve months of age when compared to those entering earlier (*P* <0.03 for trend). The number of antibiotic prescriptions in the first six years of life was 32% higher only in those children who entered daycare between six to twelve months of age (aRR for antibiotic: 1.32; 95% CI: 1.04 to 1.67).

**Table 3:** Use of healthcare resources during the first six years of life by daycare attendance

**Outcome by daycare attendance**	**Number**	**Child-years**	**Rate/100 child-years (95% CI)**	**Adjusted**^**a **^**rate ratio (95% CI)**
GP consultations for URTI and AOM				
No daycare in first year	2,053	2,424.3	84.7 (81.1 to 88.4)	1.00
Day care in first year	6,643	7,325.2	90.7 (88.5 to 92.9)	1.15 (1.00 to 1.31)
Age 0 to 2 months	926	1,104.4	83.8 (78.6 to 89.2)	1.05 (0.86 to 1.27)
Age 3 to 5 months	4,505	5,081.0	88.7 (86.1 to 91.2)	1.13 (0.98 to 1.29)
Age 6 to 2 months	1,213	1,139.8	106.4 (100.6 to 112.4)	1.32 (1.10 to 1.59)
Antibiotic prescriptions for URTI and AOM				
No daycare in first year	504	2,424.3	20.8 (19.1 to 22.6)	1.00
Day care in first year	1,517	7,325.2	20.7 (19.7 to 21.8)	1.08 (0.91 to 1.28)
Age 0 to 2 months	214	1,104.4	19.3 (17.0 to 22.0)	1.02 (0.80 to 1.29)
Age 3 to 5 months	1,012	5,081.0	19.9 (18.7 to 21.1)	1.04 (0.87 to 1.24)
Age 6 to 12 months	292	1,139.8	25.6 (22.9 to 28.6)	1.32 (1.04 to 1.67)
				**Adjusted**^**a **^**hazard ratio (95%CI)**
Referral to specialist for URTI and AOM				
No daycare in first year	47	2,424.3	1.9 (1.4 to 2.6)	1.00
Day care in first year	185	7,325.2	2.5 (2.2 to 2.9)	1.43 (1.01 to 2.03)
Age 0 to 2 months	29	1,104.4	2.6 (1.8 to 3.7)	1.41 (0.87 to 2.28)
Age 3 to 5 months	120	5,081.0	2.4 (2.0 to 2.8)	1.32 (0.92 to 1.92)
Age 6 to 12 months	36	1,139.8	3.2 (2.2 to 4.3)	1.89 (1.20 to 2.98)

^a^Adjusted for gender, parental education level, older siblings and duration of exclusive breastfeeding. AOM, acute otitis media, CI, confidence interval; GP, general practitioner; URTI, upper respiratory tract infection.

## Discussion

This study showed that the use of health care resources up to the age of six years was higher in children who entered day care in the first year of life compared to children cared for at home during that period, in spite of a similar total number of GP-diagnosed URTI and AOM episodes. This effect was most pronounced in children entering daycare between six to twelve months of age when compared to children starting at an even earlier age.

There is little doubt that daycare attendance is associated with an increased incidence of respiratory infections at preschool age (<4 years of age) [9-12,21,28]. The daycare environment provides a setting that facilitates transmission of infectious diseases. Prospective studies investigating the association between daycare attendance and infections beyond preschool age, showed a protective effect of daycare against respiratory infections in later childhood [14-16]. Our study confirms these findings to some extent, that is, children attending daycare before six months of age have lower rates of URTI and AOM after the age of four years and the cumulative incidence of URTI and AOM in the first six years was not significantly different in children who did and did not attend daycare in their first year of life. This suggests that first-year daycare attendance influences the timing of infections rather than the overall number of infections a child experiences during childhood. Notably, our study showed that the impact of URTI and AOM episodes on the child’s health in the first six years of life was substantially higher in children attending daycare as demonstrated by the increased number of GP consultations, antibiotic prescriptions and referrals among these children. These associations have, until now, only been demonstrated in preschool aged children [18-20,22,23]. Our study has demonstrated that the increased use of healthcare resources for URTI and AOM in children attending daycare during early infancy is persistent and not fully compensated by a lower use of healthcare resources during school age.

The increased use of healthcare resources in first-year daycare attendees suggests that early URTI and AOM episodes are more severe compared to later infections. Interestingly, children who enter daycare between six and twelve months of age show consistently higher healthcare resource use when compared to children entering before six months of age. A possible explanation may be the fact that children who attend daycare early in life have higher levels of maternal antibodies when being first exposed to pathogens compared to children attending daycare from six months of age [29,30]. Another explanation could be that children who enter daycare beyond six months of age had more initial health problems resulting in delayed start of daycare and that these children were, therefore, at increased risk for developing infections. We, however, think that this is not very likely since the WHISTLER population is relatively healthy; children born pre-term, with congenital abnormalities or neonatal respiratory diseases were excluded at baseline. To gain more insight into the severity of URTI and AOM in daycare attendees at different ages, additional relevant aspects of disease episodes should be investigated in future studies, such as episode duration and severity of symptoms.

The major strength of our study on the long-term effects of daycare on GP-diagnosed URTI and AOM episodes and related healthcare resource use is the large sample size and the prospective data collection. Information on URTI and AOM episodes was collected independently of exposure status, which minimizes information bias. Still some methodological limitations should be considered. Firstly, the information collected on daycare attendance did not include data on the type of daycare facility or group size. Previously, large daycare group size was found to increase infection rates at early age [8,9,28,31] and, according to Cote *et al*., protect against infections during elementary school years [15] when compared to children cared for at home. Furthermore, Morrissey *et al*. showed that children attending multiple daycare arrangements experience more respiratory problems [32]. Due to the lack of contrast in our study, the effect of daycare attendance might be underestimated for children attending large-group or multiple daycares and overestimated for small-group daycare attendees. Secondly, we cannot make inference to the long-term effects of daycare entry after the age of one year since data on daycare attendance beyond the first year of life were not available in our study. Some of the children classified in the reference group of no daycare might be exposed to daycare after their first year of life. This could lead to an underestimation of the effect of daycare. Finally, in this study, families with a high SES were overrepresented compared to the district population average. In a Swedish study, children from low SES families are less likely to attend daycare compared to children from higher SES families [18]. Indeed, the percentage of children attending daycare in this study is higher compared to the population average (approximately 80% in this study versus approximately 60% in the average Dutch population) [33]. Although this may imply selection, we consider selection bias unlikely since parents are included before the occurrence of URTI and AOM and the exposure to daycare. Furthermore, it is not plausible that the found associations between daycare attendance and the number of episodes and healthcare use for respiratory infections will differ by SES.

## Conclusions

This study showed that children who enter daycare under the age of one year have a higher use of healthcare resources for URTI and AOM in the first six years compared to children who were cared for at home. Both groups had a similar number of GP consultations over the same period; however, the timing of infections in daycare attendees was earlier in life resulting in higher use of healthcare resources than the infections which occurred later in childhood in children cared for at home. Our findings emphasize the need for improved prevention strategies in daycare facilities to lower infection rates at the early ages. Future studies are needed to determine which prevention strategies may be most effective, for example, reduction of daycare group size or determination of an optimal age of day care entry.

## Abbreviations

AOM: acute otitis media; CI: confidence interval; GEE: generalized estimating equations; GP: general practitioner; HR: hazard ratio; IRR: incidence rate ratio; SD: standard deviation; SES: socio-economic status; URTI: upper respiratory tract infection; WHISTLER: WHeezing Illnesses STudy LEidsche Rijn.

## Competing interests

All authors declare that they have no competing interests, except for AGMS who has received an educational grant from GlaxoSmithKline, The Netherlands for a study of the microbiology of acute tympanostomy tube otorrhoea.

## Authors’ contributions

MLAdH, PBV, RPV and HAS developed the concept of the present study as part of the WHISTLER study. CSPMU and CKvdE designed and supervised the WHISTLER study and obtained funding. MLAdH is guarantor. MLAdH performed the statistical analyses and all authors contributed to interpreting the results. MLAdH drafted the manuscript and all authors including AS, DB, EAMS and RAMJD critically revised it for important intellectual content. All authors had full access to all of the data (including statistical reports and tables) in the study and take responsibility for the integrity of the data and the accuracy of the data analysis. All authors read and approved the final manuscript.

## Pre-publication history

The pre-publication history for this paper can be accessed here:

http://www.biomedcentral.com/1741-7015/12/107/prepub

## References

[B1] Hak E Rovers MM Kuyvenhoven MM Schellevis FG Verheij TJ Incidence of GP-diagnosed respiratory tract infections according to age, gender and high-risk co-morbidity: the Second Dutch National Survey of General Practice Fam Pract 2006 23 291 294 16464869 10.1093/fampra/cmi121

[B2] Van Deursen AM Verheij TJ Rovers MM Veenhoven RH Groenwold RH Bonten MJ Sanders EA Trends in primary-care consultations, comorbidities, and antibiotic prescriptions for respiratory infections in The Netherlands before implementation of pneumococcal vaccines for infants Epidemiol Infect 2012 140 823 834 21781368 10.1017/S0950268811001361

[B3] Brouwer CN Rovers MM Maille AR Veenhoven RH Grobbee DE Sanders EA Schilder AG The impact of recurrent acute otitis media on the quality of life of children and their caregivers Clin Otolaryngol 2005 30 258 265 16111423 10.1111/j.1365-2273.2005.00995.x

[B4] Monasta L Ronfani L Marchetti F Montico M Vecchi Brumatti L Bavcar A Grasso D Barbiero C Tamburlini G Burden of disease caused by otitis media: systematic review and global estimates PloS One 2012 7 e36226 22558393 10.1371/journal.pone.0036226 PMC3340347

[B5] Rovers MM The burden of otitis media Vaccine 2008 26 G2 G4 19094933 10.1016/j.vaccine.2008.11.005

[B6] Schnabel E Sausenthaler S Brockow I Liese J Herbarth O Michael B Schaaf B Kramer U von Berg A Wichmann HE Heinrich J LISA Study Group Burden of otitis media and pneumonia in children up to 6 years of age: results of the LISA birth cohort Eur J Pediatr 2009 168 1251 1257 19159954 10.1007/s00431-008-0921-9

[B7] Wolleswinkel-van den Bosch JH Stolk EA Francois M Gasparini R Brosa M The health care burden and societal impact of acute otitis media in seven European countries: results of an Internet survey Vaccine 2010 28 G39 G52 21075269 10.1016/j.vaccine.2010.06.014

[B8] Marx J Osguthorpe JD Parsons G Day care and the incidence of otitis media in young children Otolaryngol Head Neck Surg 1995 112 695 699 7777354 10.1016/S0194-59989570178-8

[B9] Rovers MM Zielhuis GA Ingels K van der Wilt GJ Day-care and otitis media in young children: a critical overview Eur J Pediatr 1999 158 1 6 9950299 10.1007/pl00021272

[B10] Sun Y Sundell J Early daycare attendance increase the risk for respiratory infections and asthma of children J Asthma 2011 48 790 796 21838620 10.3109/02770903.2011.604884

[B11] Wald ER Guerra N Byers C Frequency and severity of infections in day care: three-year follow-up J Pediatr 1991 118 509 514 2007922 10.1016/s0022-3476(05)83370-0

[B12] Nafstad P Hagen JA Oie L Magnus P Jaakkola JJ Day care centers and respiratory health Pediatrics 1999 103 753 758 10103298 10.1542/peds.103.4.753

[B13] Louhiala PJ Jaakkola N Ruotsalainen R Jaakkola JJ Form of day care and respiratory infections among Finnish children Am J Public Health 1995 85 1109 1112 7625505 10.2105/ajph.85.8_pt_1.1109 PMC1615809

[B14] Ball TM Holberg CJ Aldous MB Martinez FD Wright AL Influence of attendance at day care on the common cold from birth through 13 years of age Arch Pediatr Adolesc Med 2002 156 121 126 11814371 10.1001/archpedi.156.2.121

[B15] Cote SM Petitclerc A Raynault MF Xu Q Falissard B Boivin M Tremblay RE Short- and long-term risk of infections as a function of group child care attendance: an 8-year population-based study Arch Pediatr Adolesc Med 2010 164 1132 1137 21135342 10.1001/archpediatrics.2010.216

[B16] Zutavern A Rzehak P Brockow I Schaaf B Bollrath C von Berg A Link E Kraemer U Borte M Herbarth O Wichmann HE Heinrich J LISA Study Group Day care in relation to respiratory-tract and gastrointestinal infections in a German birth cohort study Acta Paediatr 2007 96 1494 1499 17666100 10.1111/j.1651-2227.2007.00412.x

[B17] PrabhuDas M Adkins B Gans H King C Levy O Ramilo O Siegrist CA Challenges in infant immunity: implications for responses to infection and vaccines Nat Immunol 2011 12 189 194 21321588 10.1038/ni0311-189

[B18] Hjern A Haglund B Rasmussen F Rosen M Socio-economic differences in daycare arrangements and use of medical care and antibiotics in Swedish preschool children Acta Paediatr 2000 89 1250 1256 11083384 10.1080/080352500750027655

[B19] Silverstein M Sales AE Koepsell TD Health care utilization and expenditures associated with child care attendance: a nationally representative sample Pediatrics 2003 111 e371 e375 12671154 10.1542/peds.111.4.e371

[B20] Thrane N Olesen C Md JT Sondergaard C Schonheyder HC Sorensen HT Influence of day care attendance on the use of systemic antibiotics in 0- to 2-year-old children Pediatrics 2001 107 E76 11331726 10.1542/peds.107.5.e76

[B21] Brady MT Infectious disease in pediatric out-of-home child care Am J Infect Control 2005 33 276 285 15947744 10.1016/j.ajic.2004.11.007

[B22] Hedin K Andre M Hakansson A Molstad S Rodhe N Petersson C Physician consultation and antibiotic prescription in Swedish infants: population-based comparison of group daycare and home care Acta Paediatr 2007 96 1059 1063 17498187 10.1111/j.1651-2227.2007.00323.x

[B23] van de Pol AC van der Gugten AC van der Ent CK Schilder AG Benthem EM Smit HA Stellato RK de Wit NJ Damoiseaux RA Referrals for recurrent respiratory tract infections including otitis media in young children Int J Pediatr Otorhinolaryngol 2013 77 906 910 23566424 10.1016/j.ijporl.2013.03.003

[B24] Katier N Uiterwaal CS de Jong BM Kimpen JL Verheij TJ Grobbee DE Brunekreef B Numans ME van der Ent CK The Wheezing Illnesses Study Leidsche Rijn (WHISTLER): rationale and design Eur J Epidemiol 2004 19 895 903 15499901 10.1023/B:EJEP.0000040530.98310.0c PMC7087709

[B25] Verbeke M Schrans D Deroose S De Maeseneer J The International Classification of Primary Care (ICPC-2): an essential tool in the EPR of the GP Stud Health Technol Inform 2006 124 809 814 17108613

[B26] Donders AR van der Heijden GJ Stijnen T Moons KG Review: a gentle introduction to imputation of missing values J Clin Epidemiol 2006 59 1087 1091 16980149 10.1016/j.jclinepi.2006.01.014

[B27] Rubin DB Multiple Imputation for Non-Response in Surveys 1987 New York: John Wiley

[B28] National Institute of Child Health and Human Development Early Child Care Research Network Child care and common communicable illnesses: results from the National Institute of Child Health and Human Development Study of Early Child Care Arch Pediatr Adolesc Med 2001 155 481 488 11296076 10.1001/archpedi.155.4.481

[B29] Palmeira P Quinello C Silveira-Lessa AL Zago CA Carneiro-Sampaio M IgG placental transfer in healthy and pathological pregnancies Clin Dev Immunol 2012 2012 985646 22235228 10.1155/2012/985646 PMC3251916

[B30] Zinkernagel RM Maternal antibodies, childhood infections, and autoimmune diseases N Engl J Med 2001 345 1331 1335 11794153 10.1056/NEJMra012493

[B31] Bradley RH Child care and common communicable illnesses in children aged 37 to 54 months Arch Pediatr Adolesc Med 2003 157 196 200 12580692 10.1001/archpedi.157.2.196

[B32] Morrissey TW Multiple child care arrangements and common communicable illnesses in children aged 3 to 54 months Matern Child Health J 2013 17 1175 1184 22935912 10.1007/s10995-012-1125-5

[B33] Oploo van M Engelen M Tweemeting trendonderzoek kinderopvang eindrapport (Trend research in childcare) 2006 [http://www.rijksoverheid.nl/documenten-en-publicaties/rapporten/2007/05/14/tweemeting-trendonderzoek-kinderopvang.html]

